# UNOS/OPTN Data-guided Assessment of Focal Segmental Glomerulosclerosis After Kidney Transplantation and Evaluation of Immunosuppressive Protocols in a Steroid-free Center

**DOI:** 10.1097/TXD.0000000000001196

**Published:** 2021-08-05

**Authors:** Sunil M. Kurian, Samantha R. Spierling Bagsic, Jamie Case, Bethany L. Barrick, Randolph Schaffer, James C. Rice, Christopher L. Marsh

**Affiliations:** 1 Division of Organ Transplant, Scripps Center for Organ Transplantation, Scripps Clinic & Green Hospital, La Jolla, CA.; 2 Scripps Clinic Bio-Repository and Bio-Informatics Core, Scripps Clinic & Green Hospital, La Jolla, CA.; 3 Scripps Whittier Diabetes Institute, Scripps Health, San Diego, CA.

## Abstract

**Background.:**

Focal segmental glomerulosclerosis (FSGS) is a common recurrent glomerulopathy associated with graft loss and patient survival after kidney transplantation (KT). However, its natural history, clinical predictors, and treatment response are still poorly understood. Steroid withdrawal regimens in KT have been associated with improvements in cardiovascular risk and patient outcomes. The Scripps Center for Organ Transplantation (SCOT) uses a rapid low-dose steroid withdrawal immunosuppression (IS) protocol for KT maintenance.

**Methods.:**

We assessed the impact of our protocol on FSGS disease recurrence over a 10-y period to reassess our steroid and IS protocols and to evaluate if our patient outcomes diverge from published data. We compared 4 groups: steroids always, steroid free, steroid switch on, and steroid weaned off. We used IS and induction-matched retrospective data from United Network for Organ Sharing (UNOS) to investigate patient and graft survival for FSGS at SCOT.

**Results.:**

Our analysis results differ from earlier studies showing that FSGS was associated with a higher risk of graft loss, perhaps because of selection of a UNOS data set filtered to match the SCOT IS protocol for making direct comparisons. Overall outcomes of graft failure and recipient death did not differ between SCOT patients and steroid-free transplant patient data from the UNOS data for FSGS. SCOT recurrence rate for FSGS was 7.5%, which was lower than in most published single-center studies.

**Conclusions.:**

Based on our results, we believe that it is safe to continue the steroid avoidance protocols at SCOT and the steroid-free protocol may not be detrimental when the adverse effects and toxicities associated with steroid use are considered.

## INTRODUCTION

Focal segmental glomerulosclerosis (FSGS) is the leading cause of nephrotic syndrome in adults. Cohort studies show an FSGS incidence of 15%–30% across racial and ethnic groups in children and adults.^1–4^ Primary idiopathic FSGS recurs in 30%–50% of patients following kidney transplantation (KT),^5^ with other studies using stricter clinical and diagnostic definitions of FSGS putting the incidence of recurrence disease at ~11%.^6^ Steroid therapy is usually only considered for patients with idiopathic FSGS associated with nephrotic syndrome. Though recent observational studies have reported better FSGS outcomes with steroid treatment, these usually come with the risk of high doses and longer duration of steroid treatment.^7,8^ Moreover, the use of steroids in secondary FSGS is limited in current practice, and these patients are already not treated with standard immunosuppressive therapy.^9^

Steroid withdrawal regimens in KT have been promising. In a study of early steroid withdrawal, avoiding steroids was associated with improvements in cardiovascular risk factors such as triglycerides, new onset diabetes mellitus after transplantation requiring insulin, and weight gain.^10^ A regimen of tacrolimus/mycophenolate mofetil (MMF)/antibody induction therapy allowed early steroid withdrawal, with results comparable to long-term low-dose (5 mg/d) prednisone therapy. Similarly, the FRANCIA trial showed similar, noninferior 5-y efficacy profiles and reduced morbidities in steroid-free (SF) patients compared with patients who received steroids for at least 6 mo.^11^

There is some ambiguity with regards to what encompasses a “SF” regimen after transplantation. Most immunosuppression (IS) regimens classified as “SF” still give patients an initial dose of steroids, which is then either rapidly withdrawn, tapered, or discontinued. In 2 such studies, patients received steroids in tapering doses for up to 5 d,^12,13^ and another study included a group tapered off steroids only at 1 y.^13^ In a large retrospective study of IgA nephropathy recurrence using United Network for Organ Sharing (UNOS)/OPTN data, a major weakness acknowledged was the criterion used for determining “steroid use,” which was defined on the basis of follow-up data of patients still on steroids at discharge, which might not represent true longitudinal use of steroids upon follow-up.^14^ It is also difficult to establish if patients were briefly put on steroids to treat possible episodes of rejection. Similarly, it cannot be ascertained which recipients had early steroid withdrawal regimen compared with a late withdrawal as seen in some transplant centers. The 2 single-center studies mentioned earlier also differed in the use of IS^12^ or both IS and induction therapies^13^ for the comparison groups.

At the Scripps Center for Organ Transplantation (SCOT) we use a rapid low-dose steroid withdrawal IS protocol for renal transplant maintenance. Patients are given a preoperation steroid dose of 1 mg/kg followed by 1 mg/kg 6 h after antithymocyte globulin (ATG) induction followed by a dose reduction to 0.3 mg/kg postoperative day 1 and 2. The steroid dosage is also substantially lower compared with most transplant centers that begin with preoperative doses as high as 500 mg and postoperative doses of 1–2 mg/kg for several days or weeks.

Given that the SCOT is a low-dose and short-duration posttransplant steroid withdrawal center, we wanted to assess the impact of our protocol on FSGS recurrence over a 10-y period. We hypothesized that this knowledge would help us reassess and change our steroid and IS protocols if they diverge from publicly available data. Therefore, in the current study, we used retrospective data from an IS and induction (ATG induction and tacrolimus/MMF maintenance) matched set of patients from the UNOS database to investigate patient and graft survival for FSGS. We specifically assess the effects of SF regimens versus regimens that use chronic steroid maintenance. To address the potential shortcoming of the UNOS data, we looked at 2 additional groups of patients. One group was weaned off steroids sometime posttransplant and another group where steroids were initiated after an episode of kidney dysfunction such as rejection. Furthermore, we evaluated outcomes as well as disease recurrence in FSGS patients at the SCOT. We believe that the analysis of patient and graft survival of the SCOT cohort and its comparison with a general matched US renal transplant population will help us continue to evaluate the safety and efficacy of our current steroid withdrawal protocol.

## MATERIALS AND METHODS

### Human Subjects

#### Scripps Center for Organ Transplantation

The Scripps Health Institutional Review Board approved this single-center retrospective study (IRB-16-6750). Study subjects included adult (>18 y at time of transplant) recipients of a kidney-only transplant (kidney-pancreas, liver-kidney transplants were excluded). Both living donor (LD) and deceased donor transplants were included. Other exclusion criteria included pediatric patients, multiorgan transplants, retransplants, loss to follow-up, and no data on steroid regimen. All transplants took place between January 1, 2009, and December 31, 2018.

Subjects included all recipients of a renal transplant at the SCOT on an SF IS maintenance regimen. The SCOT induction therapy with 3 doses of ATG (thymoglobulin) at 2 mg/kg for a total target dose of 6 mg/kg was used in all patients. Premedication consisted of MMF (CellCept) at 3 g/d, tapered to 2 g/d after 1 wk. Tacrolimus (Prograf) was started at a low dose in the first week and increased to reach target levels of 8–10 ng/dL. A total of 40 patients with FSGS with primary diagnosis demonstrated on a renal allograft biopsy with findings of diffuse podocyte foot process effacement on electron microscopy were used for the analysis. The mean follow-up time for the SCOT cohort was 2.8 y.

#### United Network for Organ Sharing

**FIGURE 1.**
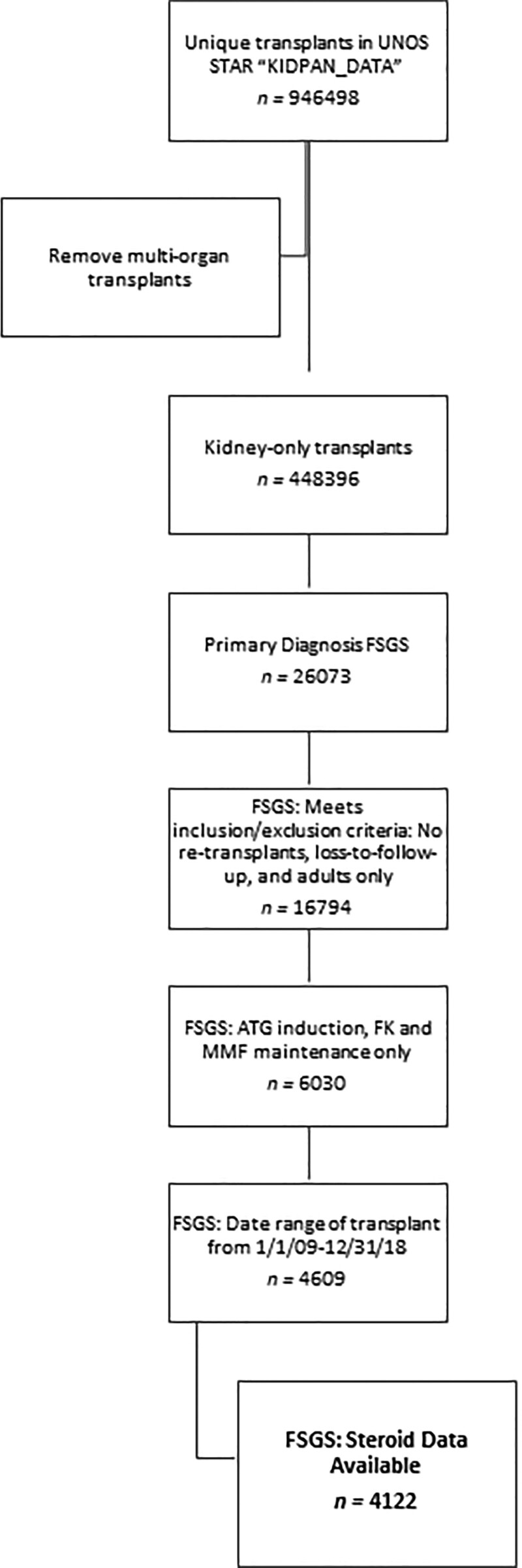
Flow schematic showing inclusion/exclusion criteria and derivation of study sample from the UNOS STAR database for diagnosis of FSGS. ATG, antithymocyte globulin; FK, FK506 (Tacrolimus); FSGS, focal segmental glomerulosclerosis; MMF, mycophenolate mofetil; UNOS, United Network for Organ Sharing.

Kidney transplant data were extracted from the UNOS database files requested by SCOT. Data for kidney-only transplants with primary diagnosis of FSGS at time of transplant were used. Patients with retransplants or status designated lost to follow-up were excluded. Only transplants that received thymoglobulin induction, tacrolimus, and CellCept maintenance were included in the analyses for a total of 4122. Steroid use was based on designated steroid maintenance at the time of discharge, as well as at all listed follow-up time points. SF indicated a subject who did not have steroid maintenance at discharge and had no indication of steroid maintenance at any follow-up. Steroid always (SA) indicated a subject received steroid maintenance at discharge and had no indication of steroid withdrawal at any follow-up. Steroid maintenance use was captured at follow-up visits, and subjects who switched steroid maintenance protocols were further grouped as steroid switch on (SSO), those who initially were not on maintenance steroids and later started taking steroids, and steroid weaned off (SWO), those initially on maintenance steroids and later taken off. If no data on steroid use were provided at follow-up, it was assumed that the steroid maintenance regimen was consistent with previous follow-up/discharge. Steroid use for acute antirejection episodes was not factored into analyses. The number of transplants analyzed is described in Figure 1. UNOS data were only included for the same dates as the SCOT data (January 1, 2009, and December 31, 2018) for direct comparisons. Recurrent FSGS was determined from the UNOS documentation and any instance of disease recurrence at any follow-up was coded as recurrent disease. The mean follow-up time for the UNOS cohort was 3.1 y.

### Statistical Analyses

Analyses comparing the 4 steroid regimens (SF, SA, SSO, and SWO) were done with either chi-square tests for categorical data, and 1-way ANOVA for normally distributed, or Kruskal-Wallis tests for nonnormally distributed continuous data. Post hoc pairwise comparisons used Holm adjustment methods. Unadjusted survival analyses were run for the outcomes of graft survival (nonfailure), overall patient survival, recurrence-free survival, and rejection-free survival using the Kaplan-Meier method using the log-rank test for significance to compare survival curves and report hazard ratios (HRs) and their confidence intervals (CIs). Post hoc comparisons between all 4 regimens were conducted using Cox proportional hazards analyses with SA treated as a reference. The statistical software R (v.3.5.3) and GraphPad Prism v.8 were used for all analyses and figure generation. All *P* values were 2-tailed and *P* values <0.05 were considered statistically significant.

## RESULTS

### UNOS Data

Of 448 396 unique kidney-only transplants listed in the UNOS registry, 26 073 (5.8%) had a primary diagnosis of FSGS. Of these, 4122 (15.8%) patients met all inclusion/exclusion criteria for the present study (Figure 1). In the UNOS data, SA maintenance accounted for 58.1% (n = 2395) of transplants, with 22.1% (n = 913) being SF, 7.2% (n = 295) being SSO, and 12.6% (n = 519) being SWO maintenance. Donors did not significantly differ in ethnicity or gender. Age differences between treatment groups were only significant in that mean age was older in SWO (mean, 38.9; SD, 14.8) compared with SA (mean, 37; SD, 14.5). Significantly more SF transplants (46.8%) were from LDs (versus deceased) compared with SA, SSO, and SWO (31.9%, 37.6%, and 32.6%, respectively; *P* < 0.001). Related LDs did not significantly differ between the groups. Deceased donors did not differ in cause of death, though extended criteria donors were more frequent, specifically with SWO (14.6%) versus SA (9.5%). There were no significant differences in organs in HLA-A, HLA-B, or HLA-DR mismatch or Kidney Donor Profile Index between all groups; however, overall HLA mismatch was greater among SA recipients compared with SF (*P* < 0.02). Cold ischemic time was significantly lower in SF recipients compared with SA *(P* < 0.001) and trended similarly for SF versus SSO and SWO (Table 1).

**TABLE 1.** FSGS donor and organ characteristics are described for SA or SF, SSO, and SWO subjects

FSGS donor/organ characteristics	SA (n = 2395)	SF (n = 913)	SSO (n = 295)	SWO (n = 519)	All group comparison *P* *
	n (%)	n (%)	n (%)	n (%)	
Living donor, n (%)	763 (31.9%)	427 (46.8%)	111 (37.6%)	169 (32.6%)	<0.001^*a,*^^*c,*^^*d*^
Related living donor, n (%)	340 (44.6%)	196 (45.9%)	53 (47.7%)	78 (46.2%)	0.908
Deceased donor cardiovascular COD, n (%)	259 (15.9%)	74 (15.2%)	28 (15.2%)	54 (15.4%)	0.983
ECD donor or not, n (%)	155 (9.5%)	48 (9.9%)	19 (10.3%)	51 (14.6%)	0.043^*b*^
Gender (male), n (%)	1373 (57.3%)	487 (53.3%)	154 (52.2%)	283 (54.5%)	0.094
Ethnicity, n (%)					0.095
White	1587 (66.3%)	626 (68.6%)	201 (68.1%)	369 (71.1%)	
Black	375 (15.7%)	124 (13.6%)	40 (13.6%)	72 (13.9%)	
Hispanic	314 (13.1%)	131 (14.4%)	35 (11.9%)	63 (12.1%)	
Other	119 (5%)	32 (3.5%)	19 (6.4%)	15 (2.9%)	
HLA-DR mismatch, n (%)					0.107
0	319 (13.4%)	142 (15.6%)	38 (12.9%)	72 (13.9%)	
1	1217 (51%)	482 (53.1%)	167 (56.6%)	258 (49.7%)	
2	852 (35.7%)	284 (31.3%)	90 (30.5%)	187 (36%)	
HLA-A mismatch, n (%)					0.101
0	297 (12.4%)	128 (14.1%)	29 (9.8%)	77 (14.8%)	
1	1023 (42.8%)	416 (45.8%)	132 (44.7%)	214 (41.2%)	
2	1068 (44.7%)	364 (40.1%)	134 (45.4%)	226 (43.5%)	
HLA-B mismatch, n (%)					0.413
0	177 (7.4%)	82 (9%)	19 (6.4%)	37 (7.1%)	
1	734 (30.7%)	300 (33%)	96 (32.5%)	162 (31.2%)	
2	1477 (61.9%)	526 (57.9%)	180 (61%)	318 (61.3%)	
HLA mismatch, median, IQR/n (%)	4 (3–5%)	4 (3–5%)	4 (3–5%)	4 (3–5%)	0.018^*a*^
0	101 (4.2%)	42 (4.6%)	7 (2.4%)	25 (4.8%)	
1	30 (1.3%)	23 (2.5%)	7 (2.4%)	14 (2.7%)	
2	162 (6.8%)	93 (10.2%)	21 (7.1%)	33 (6.4%)	
3	425 (17.8%)	153 (16.9%)	59 (20%)	85 (16.4%)	
4	575 (24.1%)	220 (24.2%)	66 (22.4%)	120 (23.1%)	
5	731 (30.6%)	264 (29.1%)	97 (32.9%)	159 (30.6%)	
6	364 (15.2%)	113 (12.4%)	38 (12.9%)	81 (15.6%)	
KDPI, mean (SD)	0.4 (0.3)	0.4 (0.3)	0.5 (0.3)	0.4 (0.3)	0.073
Age, mean (SD)	37 (14.5)	37.2 (14.3)	38.4 (14.9)	38.9 (14.8)	0.026^*b*^
Cold ischemic time, median (IQR)	12 (2.9–19.96)	8.97 (1–19)	10.56 (1.7–20.39)	10.81 (2–18.01)	<0.001^*a*^

Groups were compared by chi-square tests, 1-way ANOVA, or Kruskal-Wallis tests, and 2-tailed *P* values are shown. Post hoc comparisons were adjusted using Holm methods.

^*^Superscript indicates which pairwise comparisons are significant.

^*a*^Significant post hoc pairwise comparison between SA and SF.

^*b*^Significant post hoc pairwise comparison between SA and SWO.

^*c*^Significant post hoc pairwise comparison between SF and SSO.

^*d*^Significant post hoc pairwise comparison between SF and SWO.

^*e*^Significant post hoc pairwise comparison between SSO and SWO.

COD, cause of death; ECD, extended criteria donor; FSGS, focal segmental glomerulosclerosis; IQR, interquartile range; KDPI, Kidney Donor Profile Index; SA, steroid always; SF, steroid free; SSO, steroid switch on; SWO, steroid wean off.

Recipients did not differ in gender, age, or BMI but did significantly differ in ethnicity such that all groups differed from one another in ethnicity breakdown besides SSO and SWO, which did not differ from one another (*P* < 0.001) (see Table 2). Recipients did not differ in history of diabetes or peripheral vascular disease but did differ in history of any malignancy such that recipients in SF had significantly higher prevalence of malignancy history compared with SA (*P* = 0.005). Recipients in SF had significantly lower rates of pretransplant dialysis (*P* = 0.005) compared with SA, and among those on dialysis pretransplant, shorter median time on dialysis (*P* < 0.001) compared with SA. There were no significant differences in creatinine levels at either 1 y (±6 mo) posttransplant, but by 5 y (±6 mo), SSO recipients had higher mean creatinine levels compared with both SA and SF (Table 2).

**TABLE 2.** FSGS recipient characteristics are described for SA or SF, SSO, and SWO

FSGS recipient characteristics	SA (n = 2395)	SF) (n = 913)	SSO (n = 295)	SWO (n = 519)	All group comparison P*
Gender (male), n (%)	1421 (59.3%)	575 (63%)	173 (58.6%)	293 (56.5%)	0.085
History of any malignancy, n (%)	108 (4.5%)	69 (7.6%)	13 (4.4%)	28 (5.4%)	0.005^*a*^
Diabetes, n (%)	183 (7.6%)	80 (8.8%)	24 (8.1%)	39 (7.5%)	
Peripheral vascular disease, n (%)	90 (3.8%)	27 (3%)	10 (3.4%)	8 (1.5%)	
Ethnicity, n (%)					<0.001^*a*^^*,b,*^^*c,*^^*d,*^^*e*^
White	940 (39.3%)	468 (51.3%)	139 (47.1%)	244 (47%)	
Black	986 (41.2%)	245 (26.8%)	98 (33.2%)	194 (37.4%)	
Hispanic	303 (12.7%)	149 (16.3%)	31 (10.5%)	56 (10.8%)	
Other	166 (6.9%)	51 (5.6%)	27 (9.2%)	25 (4.8%)	
On dialysis pretransplant, n (%)	1616 (67.5%)	557 (61%)	198 (67.1%)	337 (64.9%)	0.005^*a*^
Time on dialysis pretransplant (d), median (IQR)	1420.5 (708.8–2210)	1209 (554–1940)	1248 (680–1957)	1493.7 (678.5–1944.2)	<0.001^*a*^
Age, mean (SD)	45.3 (13.9)	46.3 (14.4)	44.9 (14.6)	46.8 (14.2)	0.070
BMI, mean (SD)	28.6 (5.8)	28.5 (5.6)	28.6 (6.1)	28.6 (5.7)	0.994
Creatinine 1 y, mean (SD)	1.4 (0.5)	1.4 (0.6)	1.5 (0.6)	1.4 (0.5)	0.322
Creatinine 5 y, mean (SD)	1.4 (0.6)	1.4 (0.7)	1.6 (0.8)	1.5 (0.7)	0.001^*b,*^^*d*^
Time to first switch, median (IQR)			0.9637 (0.51–1.02)	0.9884 (0.57–1.08)	

Groups were compared by chi-square tests, 1-way ANOVA, or Kruskal-Wallis tests, and 2-tailed *P* values are shown. Post hoc comparisons were adjusted using Holm methods.

^*^Superscript indicates which pairwise comparisons are significant.

^*a*^Significant post hoc pairwise comparison between SA and SF.

^*b*^Significant post hoc pairwise comparison between SA and SSO.

^*c*^Significant post hoc pairwise comparison between SA and SWO.

^*d*^Significant post hoc pairwise comparison between SF and SSO.

^*e*^Significant post hoc pairwise comparison between SF and SWO.

^*f*^Significant post hoc pairwise comparison between SSO and SWO.

BMI, body mass index; FSGS, focal segmental glomerulosclerosis; SA, steroid always; SF, steroid free; SSO, steroid switch on; SWO, steroid wean off.

Treatment for episodes of rejection within 6 mo and 1 y were lowest in SF recipients (3.5% and 4%, respectively) compared with SA (7.3% and 8.2%, respectively) and SWO (8.4% and 11.5%, respectively). However, in SSO there was significantly higher (19.8% and 22.5%, respectively) treatment for rejection at both time points compared with all other groups (*P* < 0.001). The lowest frequency of recipients having any episodes of acute rejection over the follow-up period was SF (9.1%), followed by SA (12.2%) and SWO (17.1%), with SSO having the highest rates of recipients with any rejection episodes (34.6%) (*P* < 0.001). Few SA and SF (2.2% and 2.7%, respectively) had >1 episode of rejection, and SSO had the most patients with >1 episode of rejection (9.5%) compared with both while not significantly differing from rates in SWO (4%). Of importance in interpretation, 65.7% of SSO and 82% of SWO recipients had their first rejection episode after switching regimens (Table 3). When we looked at death with a functioning graft and all-cause death, there were significant differences in these outcomes but primarily in the SA versus SSO and SA versus SWO groups. More importantly, there were no significant differences between the SA and SF groups in terms of mortality (Table 3).

**TABLE 3.** FSGS recipient outcomes are described for SA or SF, SSO, and SWO subjects

Outcomes	SA (n = 2395)	SF (n = 913)	SSO (n = 295)	SWO (n = 519)	All group comparison P *
	n (%)	n (%)	n (%)	n (%)	
Treated for rejection w/in 6 mo					<0.001^*a,*^^*b,*^^*d,*^^*e,*^^*f*^
Yes	152 (7.3%)	28 (3.5%)	52 (19.8%)	37 (8.4%)	
No	1933 (92.7%)	781 (96.5%)	210 (80.2%)	406 (91.6%)	
Treated for rejection w/in 1 y					<0.001^*a,*^^*b,*^^*d,*^^*e,*^^*f*^
Yes	171 (8.2%)	32 (4%)	59 (22.5%)	51 (11.5%)	
No	1774 (85.1%)	749 (92.6%)	191 (72.9%)	384 (86.7%)	
Graft failure (up to 10 y)	185 (7.7%)	50 (5.5%)	50 (16.9%)	66 (12.7%)	<0.001^*b,*^^*c*^^*,d,*^^*e*^
Cause of failure					0.083
Recurrent disease	46 (24.9%)	12 (24%)	7 (14%)	14 (21.2%)	
Acute/chronic rejection	98 (53%)	18 (36%)	25 (50%)	35 (53%)	
Other/unknown	40 (21.6%)	20 (40%)	18 (36%)	17 (25.8%)	
Death with functioning graft	81 (3.4%)	48 (5.3%)	15 (5.1%)	35 (6.7%)	0.002^*c*^
Death (all cause)	94 (3.9%)	53 (5.8%)	22 (7.5%)	42 (8.1%)	<0.001^*b,*^^*c*^
Primary graft failure, recurrent disease, and renal failure	2 (0.1%)	0 (0%)	0 (0%)	1 (0.2%)	
Infection	15 (0.6%)	8 (0.9%)	2 (0.7%)	6 (1.2%)	
Cardiovascular, cerebrovascular, and respiratory	22 (0.9%)	17 (1.9%)	10 (3.4%)	10 (1.9%)	
Malignancy	12 (0.5%)	11 (1.2%)	3 (1%)	6 (1.2%)	
Other or unknown	42 (1.8%)	17 (1.9%)	6 (2%)	18 (3.5%)	
Any episodes of acute rejection?	293 (12.2%)	83 (9.1%)	102 (34.6%)	89 (17.1%)	<0.001^*a*^^*,b*^^*,c,*^^*d*^^*,e,*^^*f*^
More than 1 episode of rejection	53 (2.2%)	25 (2.7%)	28 (9.5%)	21 (4%)	<0.001^*b,*^^*c,*^^*d,*^^*f*^
Disease recurrence	161 (6.7%)	41 (4.5%)	33 (11.2%)	42 (8.1%)	<0.001^*b*,^^*d*,^^*e*^
First rejection episode after switch			67 (65.7%)	73 (82.0%)	

Groups were compared by chi-square tests, 1-way ANOVA, or Kruskal-Wallis tests, and 2-tailed *P* values are shown. Post hoc comparisons were adjusted using Holm methods.

^*^Pairwise comparisons that are significant.

^*a*^Significant post hoc pairwise comparison between SA and SF.

^*b*^Significant post hoc pairwise comparison between SA and SSO.

^*c*^Significant post hoc pairwise comparison between SA and SWO.

^*d*^Significant post hoc pairwise comparison between SF and SSO.

^*e*^Significant post hoc pairwise comparison between SF and SWO.

^*f*^Significant post hoc pairwise comparison between SSO and SWO.

FSGS, focal segmental glomerulosclerosis; SA, steroid always; SF, steroid free; SSO, steroid switch on; SWO, steroid weaned off.

**FIGURE 2.**
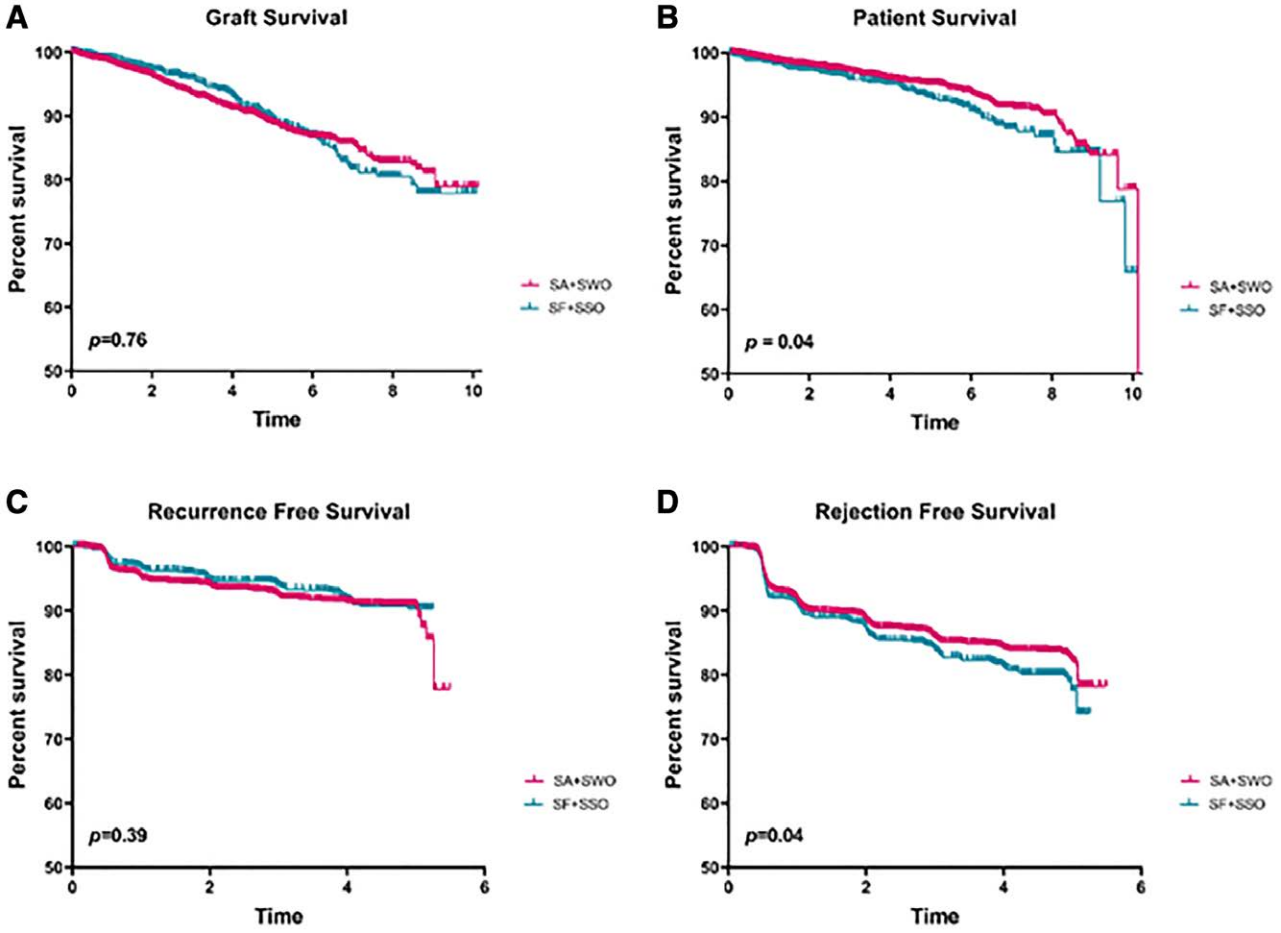
FSGS graft survival (A), overall survival (B), recurrence-free survival (C), and rejection-free survival (D) are shown among those discharged from transplant on steroids (SA + SWO) and those not discharged on steroids (SF + SSO) groups. Groups were compared by the log-rank method and *P* values are shown for each. FSGS, focal segmental glomerulosclerosis; SA, steroid always; SF, steroid free; SSO, steroid switch on; SWO, steroid wean off.

Analyzing rates of graft survival, overall survival, recurrence-free survival, and rejection-free survival between groups based on initial discharge status (steroids: SA + SWO or no steroids: SF + SSO) revealed no differences in graft or recurrence-free survival. However, poorer overall survival (HR, 1.35; 95% CI, 1.00–1.81; *P* = 0.04) and rejection-free survival (HR, 1.20; 95% CI, 1.00–1.44; *P* = 0.04) were found for SF + SSO relative to SA + SWO (Figure 2A–D).

**FIGURE 3.**
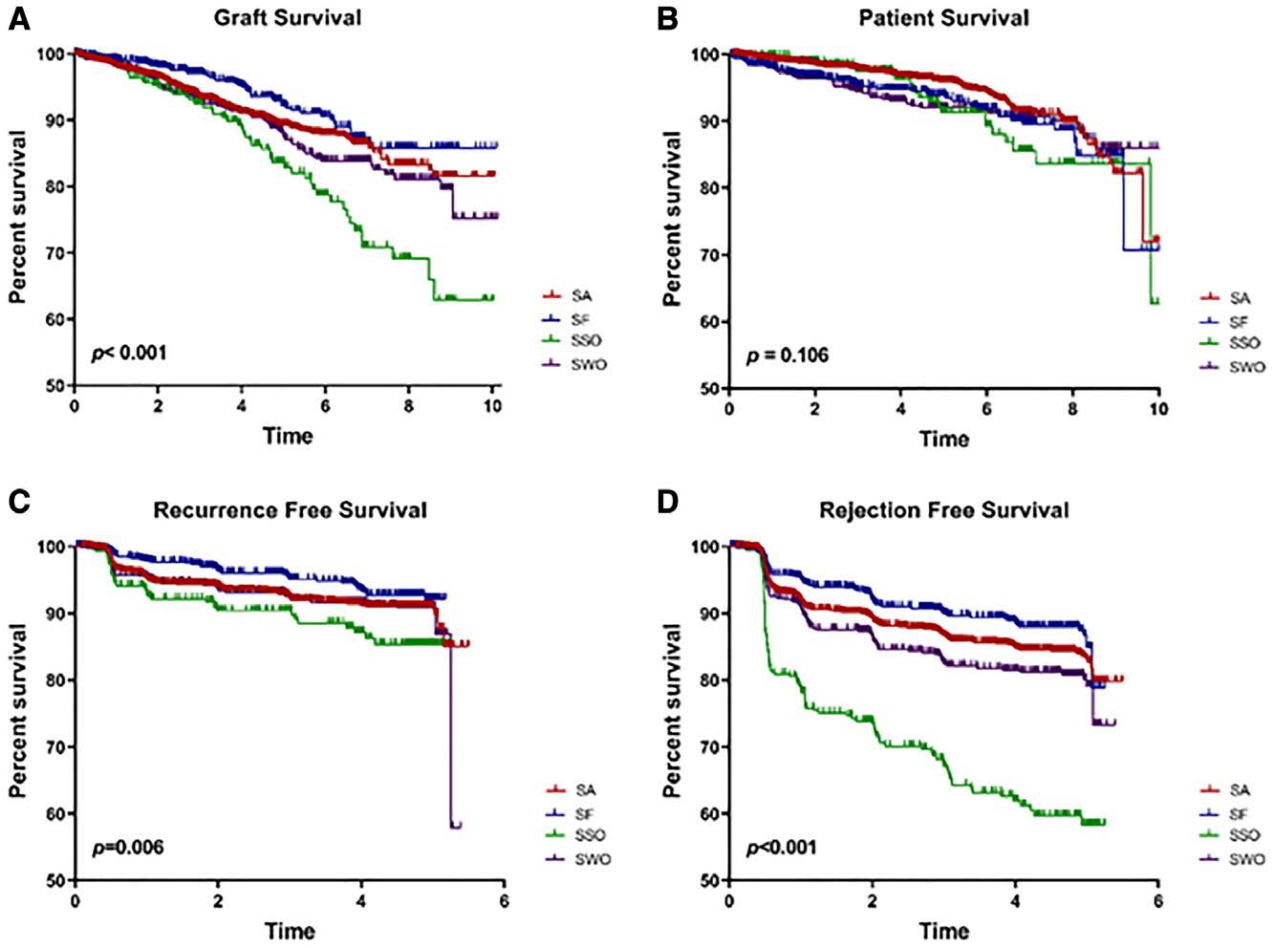
FSGS graft survival (A), overall survival (B), recurrence-free survival (C), and rejection-free survival (D) are shown among all 4 steroid regimen groups: SA, SF, SSO, and SWO. Groups were compared by the log-rank method and *P* values are shown for each. FSGS, focal segmental glomerulosclerosis; SA, steroid always; SF, steroid free; SSO, steroid switch on; SWO, steroid wean off.

**FIGURE 4.**
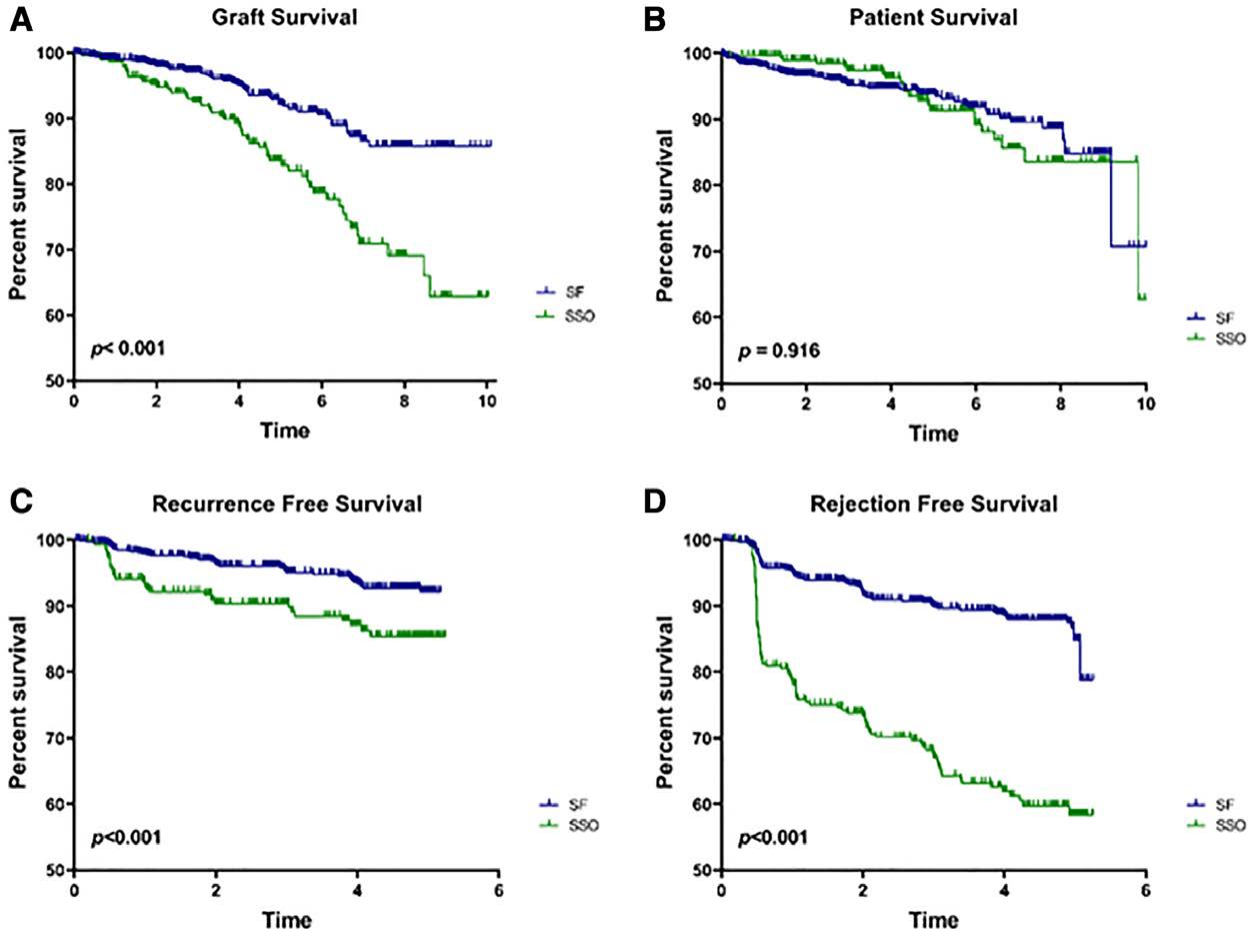
FSGS graft survival (A), overall survival (B), recurrence-free survival (C), and rejection-free survival (D) are shown for SF and SSO only. Groups were compared by the log-rank method and *P* values are shown for each. FSGS, focal segmental glomerulosclerosis; SF, steroid free; SSO, steroid switch on.

Graft failure rates were low among SF (5.5%) and SA (7.7%), not significant, but rates in each of these regimens were significantly lower than SSO (16.9%) and SWO (12.7%) (*P* < 0.001). Causes of graft failure did not significantly differ across regimens (Figure 3A–D). Graft survival differed among groups (*P* < 0.001) and was significantly improved in SF relative to SA (HR, 0.69; 95% CI, 0.51–0.95) but worse in SSO relative to SA (HR, 1.75; 95% CI,1.28–2.40). Overall recipient survival did not significantly differ between groups (*P* = 0.106), but recurrence-free and rejection-free survival did differ when all groups were compared (*P* < 0.006 and *P* < 0.0001, respectively). Relative to SA, SF had improved recurrence-free (HR, 0.67; 95% CI, 0.48–0.95) and rejection-free (HR, 0.75; 95% CI, 0.58–0.95) survival, whereas SSO had poorer recurrence-free (HR, 1.52; 95% CI, 1.04–2.21) and rejection-free (HR, 2.88; 95% CI, 2.30–3.61) survival. Because both SF and SSO recipients were immediately SF following transplant, further comparisons examined the outcomes in these groups only and confirmed no difference in patient survival, but poorer graft, recurrence-free, and rejection-free survival in SSO versus SF patients (*P* < 0.001) (Figure 4A–D).

### SCOT Data

FSGS KTs did not significantly differ from SF transplants in the UNOS database in proportion of LD, related LD, donor gender, or recipient genders, nor in donor age, recipient age, ethnicity, or cold ischemic time (*P* > 0.05). The recurrence rate of FSGS at SCOT is 7.5% and not significantly different from the UNOS data FSGS rate. Overall outcomes of graft failure and recipient death did not differ between the SCOT patients and SF transplants from the UNOS database (*P* > 0.5) for FSGS (Table 4).

**TABLE 4.** FSGS donor, organ, and recipient characteristics as well as outcomes are described for SCOT patients and the steroid-free subjects in the UNOS database

	SCOT (N = 40)	UNOS SF (N = 913)	*P*
FSGS donor/organ characteristics			
Living donor, n (%)	15 (37.5%)	427 (46.8%)	0.250
Related living donor, n (%)	7 (46.7%)	196 (45.9%)	0.953
Gender (male), n (%)	22 (55%)	487 (53.3%)	0.837
Age, mean (SD)	40.4 (14.6)	37.2 (14.3)	0.212
Cold ischemic time, median (IQR)	8.7 (20.7)	9.0 (18)	0.907
FSGS recipient characteristics			
Gender (male), n (%)	21 (52.5%)	575 (63%)	0.180
Ethnicity, n (%)			0.259
White	17 (42.5%)	468 (51.3%)	
Black	9 (22.5%)	245 (26.8%)	
Hispanic	10 (25%)	149 (16.3%)	
Asian	4 (10%)	42 (4.6%)	
Other	0 (0%)	9 (1%)	
Age, mean (SD)	48 (14.2)	46.3 (14.4)	0.4685
FSGS outcomes			
Recurrence, n (%)	3 (7.5%)	41 (4.5%)	0.615
Graft failures, n (%)	2 (5%)	50 (5.5%)	>0. 999
Death, n (%)	1 (2.5%)	53 (5.8%)	0.592

Groups were compared by chi-square tests, *t* tests, or Mann-Whitney *U* tests, as shown. Two-tailed *P* values are shown.

FSGS, focal segmental glomerulosclerosis; IQR, interquartile range; SCOT, Scripps Center for Organ Transplantation; SF, steroid free; UNOS, United Network for Organ Sharing.

## DISCUSSION

Using UNOS data, we analyzed 4122 transplants with a primary diagnosis of FSGS. Our analysis was limited to data from transplant recipients who received thymoglobulin induction as well as tacrolimus and CellCept as maintenance IS for the ease of comparison with the current SCOT IS regimen. When we compared the cumulative incidence of FSGS recurrence at the SCOT, the rates were lower than most reported studies, which ranged from ~11% to 30%. Among the patients who had recurrent FSGS, the SCOT graft loss was comparable (5%) with rates in UNOS data (5.5%) but lower than that observed in single-center studies in the existing literature that ranged from 20% to 48%.^6,15,16^

There were more SF transplants from LD (versus deceased) compared with all other groups. This agrees with literature showing that transplant protocols with rapid discontinuation of steroids in LD do not increase rejection or graft loss and that steroid withdrawal is increasingly common among LD recipients.^17,18^ Our FSGS analysis showed that SF patients have significantly better graft survival but inferior overall patient survival, though this difference was not significant between SF versus SA. This suggests that being SF may be renoprotective and preserve kidney function, but decreased survival was mainly because of malignancy-related deaths. One can hypothesize that patients on steroids may benefit from their beneficial effects on cancer prevention because glucocorticoids are often used to treat hematopoietic malignancies of the lymphoid lineage in protocols to induce cell apoptosis,^19^ and glucocorticoids are also used as cotherapy during chemotherapy to reduce side effects.^20^ Additionally, patients who are on SF IS may have been identified as having potential risk for steroid-related complications, and this may have introduced a selection bias because SF patients may already be at higher risk for mortality. This agrees with our finding that SF had significantly higher prevalence of malignancy history compared with SA. The SF group had the lowest frequency of recipients having any episodes of acute rejection over the follow-up period and surprisingly, the majority of SSO and SWO recipients had their first rejection episode after switching regimens. Even though this seems counterintuitive given that cessation of corticosteroids may increase risk of short-term rejection, a recent large clinical trial comparing corticosteroid versus no corticosteroid in kidney transplants showed that outcomes or rejection episodes did not differ significantly in either group.^21^ However, it is important to acknowledge a possible bias because of missing or incomplete data on acute rejections and steroid use at follow-up encounters, which is a limitation of both UNOS data. More importantly, all steroid regimens were based on those deemed for “maintenance” only, and acute steroid use for episodes of rejection was not examined in the present study. Such granular data on short-term steroid use are unfortunately not well documented in the UNOS database, which is a shortcoming that has been addressed by us and others.^14,22^ Our results also show that graft failure rates were highest in the SSO and SWO groups suggesting that switching steroids in IS regimes may be the most detrimental with regard to long-term outcomes.

Rapid discontinuation of steroids has been shown to be associated with a higher risk of recurrence for types of glomerulonephritis; however, the graft and patient survival were similar in SF and steroid continuation groups.^12^ In a 2006 study, there were no adverse effects in terms of increased acute rejection or graft survival for recipients who received their transplants for glomerulonephritic diseases and were also on prednisone-free maintenance IS.^23^ Their data also showed that the glomerulonephritis group that was SF showed better graft and overall survival in LD and deceased donors, though the results were not significant. The steroid withdrawal regimen at SCOT did not significantly increase FSGS recurrence rates reassuring us that the protocols were safe.

Our observation of lower FSGS recurrence risk may differ with other studies but may be attributed to our study being limited to data from UNOS on only patients who were on tacrolimus and CellCept IS and ATG induction, which may be a superior regimen. This was done to facilitate direct comparisons with the data from the SCOT. Another limitation of our study was, although the data we obtained from UNOS/OPTN were extensive, they did not have a lot of granularity and the amount of missing data and data lost to follow-up could have affected the outcomes that we addressed. This was especially true with regard to data regarding continuation of steroids because we used the steroid status upon discharge as an endpoint for our outcome analysis. This approach may lack the finer details of steroid use in between follow-up for rejection or dysfunction episodes and longer intervals between subsequent follow-up visits make this more daunting. Because we excluded subjects who switched steroid maintenance protocols, we were limited in the numbers for the final analysis, and this may explain some of the finer discrepancies between our study and other similar studies.

In conclusion, the data from the SCOT revealed that FSGS recurrence rates were consistent with the current literature and the UNOS data. Our data suggested that SCOT overall patient survival compared with the SF patients in the UNOS data was not significant. The SCOT data analysis and comparison with the UNOS data therefore make us optimistic that it is safe to continue with the SF protocols that are currently the standard of practice at the SCOT. However, we are aware of the cautionary note that SF patients tend to have higher mortality, though not significantly, which will be monitored closely at SCOT moving forward. Our data suggest that in our center, the benefits of being SF may outweigh the potential risks to patients given the adverse effects and toxicities associated with steroid use.

## ACKNOWLEDGMENTS

The authors would like to thank Dr Dianne McKay and Dr Jonathan Fisher for their comments and suggestions to improve this article. The authors would like to acknowledge the Fred and Betty Farago Research Fund for providing partial funding of this work. The authors also acknowledge the National Center for Advancing Translational Sciences of the National Institutes of Health Award number UL1TR002550.
